# Longitudinal, transcranial measurement of functional activation in the rat brain by diffuse correlation spectroscopy

**DOI:** 10.1117/1.NPh.4.4.045006

**Published:** 2017-12-05

**Authors:** Igor Blanco, Peyman Zirak, Tanja Dragojević, Clara Castellvi, Turgut Durduran, Carles Justicia

**Affiliations:** a ICFO-Institut de Ciències Fotòniques, Barcelona Institute of Science and Technology, Castelldefels, Barcelona, Spain; b Insitut d’Investigacions Biomèdiques de Barcelona (IIBB), Consejo Superior de Investigaciones Científicas (CSIC), Department of Brain Ischemia and Neurodegeneration, Barcelona, Spain; c Institut d’Investigacions Biomèdiques August Pi i Sunyer (IDIBAPS), Àrea de Neurociències, Barcelona, Spain; d Institució Catalana de Recerca i Estudis Avançats (ICREA), Barcelona, Spain

**Keywords:** blood or tissue constituent monitoring, functional monitoring and imaging, spectroscopy, speckle

## Abstract

Neural activity is an important biomarker for the presence of neurodegenerative diseases, cerebrovascular alterations, and brain trauma; furthermore, it is a surrogate marker for treatment effects. These pathologies may occur and evolve in a long time-period, thus, noninvasive, transcutaneous techniques are necessary to allow a longitudinal follow-up. In the present work, we have customized noninvasive, transcutaneous, diffuse correlation spectroscopy (DCS) to localize changes in cerebral blood flow (CBF) induced by neural activity. We were able to detect changes in CBF in the somatosensory cortex by using a model of electrical forepaw stimulation in rats. The suitability of DCS measurements for longitudinal monitoring was demonstrated by performing multiple sessions with the same animals at different ages (from 6 to 18 months). In addition, functional DCS has been cross-validated by comparison with functional magnetic resonance imaging (fMRI) in the same animals in a subset of the time-points. The overall results obtained with transcutaneous DCS demonstrates that it can be utilized in longitudinal studies safely and reproducibly to locate changes in CBF induced by neural activity in the small animal brain.

## 1 Introduction

Alterations in neuronal activity drive local changes in blood flow in order to increase blood supply to the metabolically active regions.^1^ The neurovascular coupling is one of the primary biomarkers utilized in the study of functional brain activity.

Functional neuroimaging techniques are especially interesting for the study of the progression of different pathologies^2^^–^^6^ affecting the central nervous system, as well as different mechanisms of recovery, for instance, therapy-potentiated functional recovery.^7^ This requires systems that do not interfere with the progression of either the pathology or the possible therapies, and allow the same individuals to be studied over extended periods.

The most common noninvasive neuroimaging method is functional magnetic resonance imaging (fMRI). fMRI is based on the blood oxygen level dependent (BOLD) signal,^8^ which is associated with deoxyhemoglobin concentration. Unfortunately, changes in the BOLD signal can be influenced by vascular architecture, intrinsic hemodynamic responses, and other factors, such as spin density, volume fraction, the levels of different neurotransmitters, and perfusion.^9^ Even though fMRI is considered as one of the most applicable techniques for the study of neuronal activity, it has some drawbacks, such as availability, cost, and robustness.

On the other hand, there are optical imaging modalities that are relatively inexpensive, which do not need a special infrastructure or use of an exogenous contrast. Optical techniques can provide advantages in the study of the functional activation in the brain by using intrinsic changes that can detect variations in fluorescence light, absorption, or light scattering. Techniques, such as optical intrinsic signal imaging (OISI), functional near-infrared spectroscopy, or photoacoustics study changes in the blood volume and blood oxygen saturation due to changes in the concentration of oxy- and deoxyhemoglobin induced by the neuronal activity.^10^^–^^13^ Indirect information about cerebral blood flow (CBF) can be obtained by looking at the changes of the blood volume over time by using contrast agents. There are optical techniques that directly measure blood flow, such as diffuse correlation spectroscopy (DCS), laser speckle imaging (LSI), and laser Doppler flowmetry (LDF).^14^^–^^18^ Other MRI modalities, such as cerebral blood volume (CBV)-weighted fMRI, where an intravascular contrast agent is necessary to monitor CBV changes induced by local neural activity, or arterial spin labeling (ASL), that localizes local changes in CBF, have a relatively low temporal resolution and low signal-to-noise ratio (SNR).^19^

Optical techniques are often used for imaging the hemodynamic response to different functional stimuli in the small animal brain. LDF is used to quantify the blood flow changes during the stimulation in small animal models, however, it is limited to point and superficial tissue measurements (<1  mm).^20^^–^^22^ Another technique that is often used is LSI. LSI provides two dimensional images and can measure absolute blood flow,^23^^,^^24^ but it is also limited to superficial imaging (<1  mm) of hemodynamics.^16^^,^^25^^,^^26^ The downside of both LDF and LSI is that it is necessary to clear, remove, or thin the skull depending on the species.^23^^,^^25^ On the other hand, diffuse optical techniques, such as near-infrared spectroscopy (NIRS), DCS,^27^ and speckle contrast optical tomography (SCOT)^28^ allow noninvasive measurements of the cerebral hemodynamics in the deep tissues.^12^^,^^25^^,^^29^^,^^30^ Both NIRS and DCS have been used for measurement of rodent brain transcranially^31^ with scalp retraction^27^^,^^28^ and with implanted probes in mice heads for longitudinal studies.^32^

In the present study, we combine a mild sedative with transcranial DCS to measure CBF in longitudinal (over months) brain functional activation due to forepaw stimulation and to cross-validate the activation pattern with results obtained by fMRI in the same rats by employing the routinely used BOLD signal.

## 2 Methods

All experimental procedures involving the use of laboratory animals have been carried out following the ARRIVE guidelines, the local and European legislation, and have been approved by the Ethical Committee of Animal Experimentation (approval number 7883) of the Generalitat de Catalunya.

**Fig. 1:**
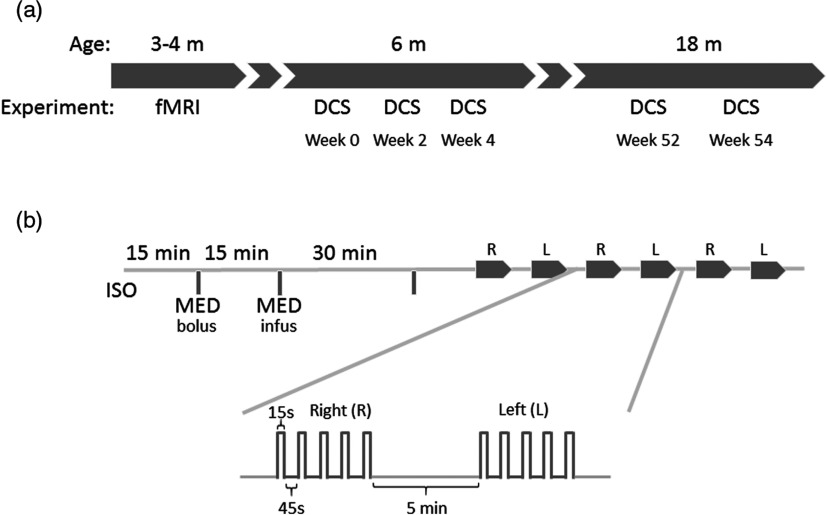
(a) Timing of the experimental procedures. (b) Experimental protocol for induction of anesthesia and stimulation session. Fifteen minutes under isoflurane (ISO) were dedicated to positioning the rats’ heads on the stereotaxic frame (or on the cradle in the fMRI experiments). Fifteen minutes after medetomidine bolus injection (MED), the infusion started. Thirty minutes were allowed for sedative stabilization before the first electrical stimulation. Each session consisted in six consecutive experiments, alternating right and left forepaw stimulation. Rectangular pulses (0.3 ms; 6 Hz; 2 mA) were used in five blocks (15-s activation and 45-s resting period each), and 5 minutes were left for recovery between experiments.

Eleven male Wistar rats with a body weight of (320±50)  g at the beginning of the experiment (3 to 4 months old) were initially used for fMRI, and the same rats were measured with DCS at the age of 6 and 18 months. Six DCS experiments were carried out at the age of 6 months, with a resting period of 15 days between sessions, and an additional four experiments were done with the same rats at the age of 18 months [Fig. 1(a)].

### 2.1 Anesthesia

Rats were anesthetized with 4% isoflurane in $O_{2}:N_{2}O$ (30:70). Animals were placed in a stereotaxic frame (or the MRI cradle in the fMRI experiments) with a face mask delivering the same gas mixture with 1.5% isoflurane, and a subcutaneous bolus (0.05  mg/Kg) of medetomidine (Domtor, Pfizer) was injected. Isoflurane was slowly discontinued over the course of the next 15 min at a rate of 0.25% every 1 min starting 3 min after the bolus. At this point, a continuous subcutaneous infusion of medetomidine (1  ml/h and 0.1  mg/Kg) was initiated for the whole duration of the experiment.

The scalp of the rat was shaved using an electric razor and depilatory cream to avoid side effects in the optical signal produced by fur. Two subdermal electrode needles were inserted in each forepaw for stimulation. Respiration rate (number of breaths per minute) was constantly registered and the body temperature was monitored with a rectal probe and maintained at (37±0.5)°C with a feedback controlled electrical blanket. Stimulation experiments started 30 min after isoflurane was discontinued and rats presented a stable condition with a respiration rate of ∼40% to 50% compared to the initial values.

Once the experiment was concluded, animals received an intraperitoneal injection (0.1  mg/Kg) of atipemazole (Antisedan, Pfizer) to reverse the effect of medetomidine.

### 2.2 Electrical Forepaw Stimulation

Forepaw stimulation consisted in rectangular pulses (2.0 mA, 6 Hz, 0.3 ms) in a paradigm of five consecutive blocks of 15-s stimulation followed by 45 s of recovery [Fig. 1(b)] with a total time of 5 minutes. Functional activation monitoring was conducted alternately three times for each forepaw. A resting period of 5 min was allowed between forepaw stimulation experiments [Fig. 1(b)].

### 2.3 Functional Magnetic Resonance Imaging

The fMRI experiments were conducted on a 7.0 T BioSpec 70/30 horizontal scanner (Bruker BioSpin, Ettlingen, Germany) equipped with an actively shielded gradient system (400  mT/m, 12-cm inner diameter). The receiver coil was a four-channel phased-array surface coil for the rat brain. Functional activation imaging was achieved with BOLD contrast MRI. Coronal multislice spin-echo (SE) EPI images were acquired using the following parameters: TE/TR=30=3000  ms; BW=150  kHz; five consecutive slices of 2 mm thickness; $\text{field-of-view}=2.56\times 2.56\;{\mathrm{cm}}^{2}$; matrix of 64×64  pixels.

#### 2.3.1 Data analysis

Statistical parametric activation maps from BOLD fMRI were constructed with the software STIMULATE.^33^ The time course was examined for each pixel during forepaw stimulation using a paired Student’s t-test with p<0.01 as the significance level, where only statically significant pixels were used to calculate median BOLD signal intensity [ΔSI BOLD (%)].

fMRI maps were calculated without any previous filtering or signal baseline thresholding. Clustering was done considering at least four adjacent activated pixels.

DCS signal was normalized to the 5-min baseline prior to the corresponding stimulation experiment.

Statistical analyses were conducted using GraphPad Prism v4.0. All data are presented as (mean±SEM)%, where SEM is the standard error of the mean. To check if the ΔSI BOLD signal and ΔrCBF are correlated, we have carried out a linear regression analysis and calculated the squared Pearson’s correlation coefficient ($r^{2}$).

### 2.4 Diffuse Correlation Spectroscopy

#### 2.4.1 Optical method

DCS is a noninvasive diffuse optical method that quantifies deep tissues blood flow. DCS measures temporal speckle fluctuations of the scattered light that are sensitive to the movement of the scattering particles, such as red blood cells.^14^^,^^34^ The dynamics of the scatterers is determined by the measurement of the intensity autocorrelation function, from which the normalized electric autocorrelation function is derived. This is then fitted with the solution of the correlation diffusion equation for the semi-infinite geometry to obtain the effective, scattering number weighted Brownian motion coefficient ($D_{B}$), which was shown to be proportional to the blood flow index (BFI).^14^^,^^35^^,^^36^ The relative cerebral blood flow (rCBF) is then obtained by normalizing the measured BFI with a given baseline.^37^

#### 2.4.2 Optical instrumentation

Light was injected into the tissue by using a long coherence laser source at 785 nm (120 mW, Crystalaser, Reno, Nevada) through a multimode fiber. The light was collected using eight single-mode fibers and sent into two arrays of four single-photon counting avalanche photodiodes (SPCM-AQ4C, Dumberry, Vaudreuil, Canada) conforming eight (2×4) detectors. The output of each detector was used to build the normalized intensity autocorrelation function by using an eight-channel correlator (Correlator.com, New Jersey).

#### 2.4.3 Diffuse correlation spectroscopy probe

**Fig. 2:**
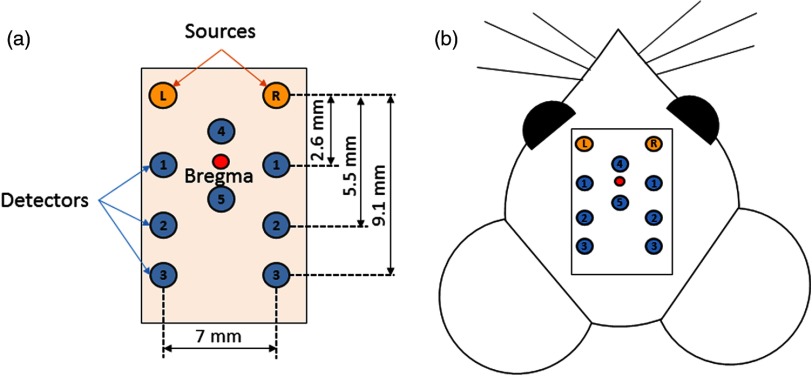
(a) A schematic of the transcutaneous probe and its design with the source–detector separations and (b) its placement on the rat head.

The DCS probe consisted of eight detectors and two source fibers (Fig. 2), with three source–detector separations 2.6, 5.5, and 9.1 mm, respectively. The probe was placed in a holder that was designed to be merged with the manipulator arm of the stereotaxic frame, ensuring a sufficiently precise positioning and good contact between the head of the rat and the transcutaneous probe. External landmarks (eyes, ears, and nose) were used for the orientation and the placement of the probe since the skull and scalp were intact, the Bregma was not visible and could not be used as a reference point.

## 3 Results

The body temperature was kept in the physiological range of (37.4±0.2)°C under the isoflurane and at (37.1±0.6)°C with medetomidine while the respiratory rate decreased during the experimental procedure when using medetomidine as a sedative agent. The initial respiratory rate, which, under isoflurane, was (87±21)  bpm dropped to (55±12)  bpm after shifting to medetomidine sedation and remained stable for the next 1.5 to 2 h increasing gradually thereafter. This temporal window was considered as the stable period to carry out the stimulation experiments.

### 3.1 Functional Magnetic Resonance Imaging

All rats (n=11) were initially subjected to fMRI and showed neuronal activation in the primary somatosensory cortex during unilateral electrical forepaw stimulation.

**Fig. 3:**
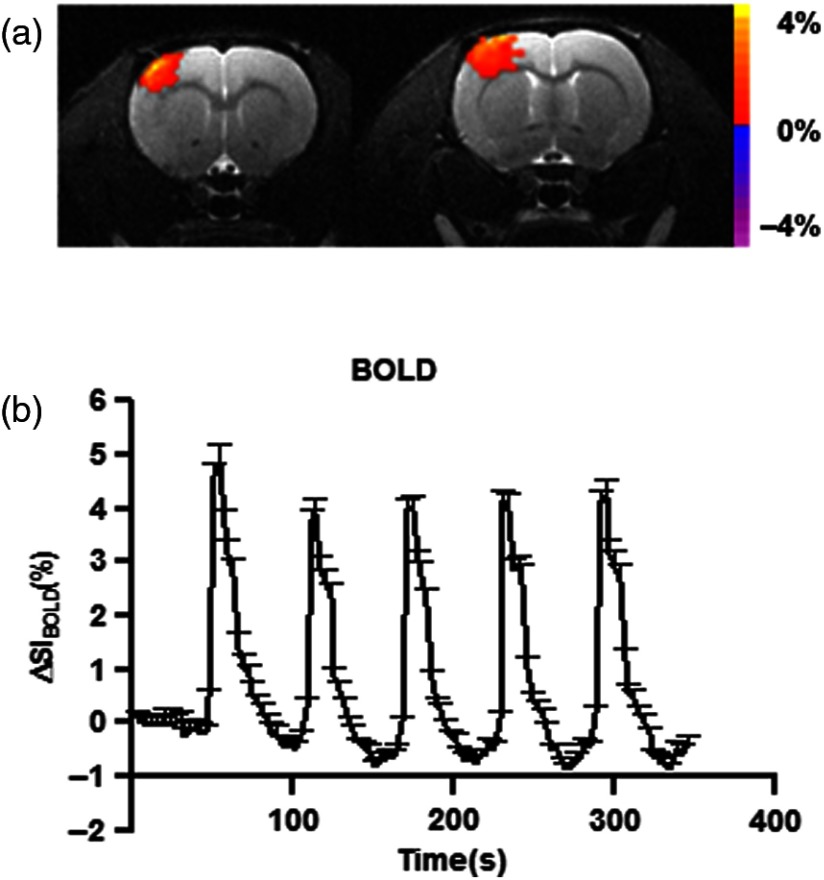
(a) BOLD fMRI activation map during right forepaw stimulation overlapped on the anatomical T2-w images. The activated region corresponds to the forelimb area of the somatosensory cortex (SIfl). (b) BOLD signal increase averaged from all rats (six experiments each). Change in signal increase was (4.2±1.6)% [(mean±SEM)%].

In Fig. 3(a), we show an example of the activation map of one animal stimulated in the right forepaw. The color map shows the activation on the cortical somatosensory region (SIfl). Averaged BOLD signal increase (%), of the 11 animals over all six experiments (three left and three right forepaw stimulation), is shown in Fig. 3(b), resulting in a mean BOLD signal increase of (4.3±1.1)%, where the profile of five consecutive time-series of activation periods composed in each experiment can be observed. In Table 1, we show data from all the animals.

**Table 1:** Relative rCBF from all animals at 6 months of age (week 0, week 2, and week 4) and at 18 months of age (week 52 and week 54) presented as ΔrCBF (%) (mean±SEM). fMRI results are presented as [$\Delta {\mathrm{SI}}_{\text{BOLD}}$ (%)].

Technique	fMRI	DCS						
Animal	ΔSI BOLD (%)	ΔrCBF (%)						
	Week 0	Week 0	Week 2	Week 4	Grouped (6 months)	Week 52	Week 54	Grouped (18 months)
Rat 1	4.3±1.3	22.8±3.2	21.9±4.8	20.2±3.6	21.6±2.9	18.4±3.6	16.3±3.7	17.3±3.1
Rat 2	3.9±0.8	17.0±4.3	16.1±4.9	15.9±3.4	16.2±3.5	16.1±3.3	14.2±3.7	15.1±2.9
Rat 3	4.5±2.0	27.8±4.4	24.7±2.6	25.9±5.5	26.1±4.1	23.6±4.2	21.0±3.4	22.2±1.7
Rat 4	4.0±0.8	20.4±3.9	20.9±2.8	23.4±4.7	21.6±1.5	21.2±4.7	16.9±3.0	19.0±2.2
Rat 5	4.5±1.2	22.4±3.8	27.9±5.8	26.7±6.1	25.6±5.2	25.8±5.9	24.6±5.4	25.2±5.6
Rat 6	4.2±1.2	22.4±4.6	16.3±3.8	24.7±5.6	21.1±3.7	19.5±2.8	17.1±3.7	18.2±2.1
Rat 7	4.1±0.4	20.3±4.0	16.7±3.4	17.2±3.7	18.0±3.2	19.3±3.9	17.4±2.6	18.3±3.3
Rat 8	4.6±0.7	25.6±4.2	20.4±3.8	23.7±5.0	23.2±1.9	23.2±4.4	19.1±3.4	21.1±1.8

### 3.2 Diffuse Correlation Spectroscopy

After the administration of the medetomidine bolus, and the discontinuation of isoflurane, the corneal and tail-pinch reflexes were absent, except in two animals, in which medetomidine did not cause a sedative effect, probably due to a high level of stress, and, therefore, were excluded. Furthermore, one animal died due to respiratory difficulties. After excluding these animals, a total of eight rats completed all the five sessions of DCS measurements.

**Fig. 4:**
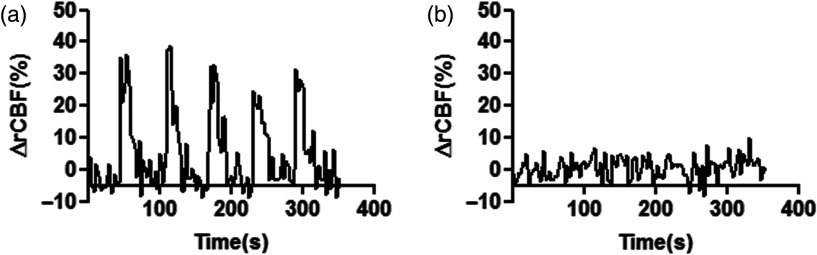
The stimulation pattern from the cortex: (a) contralateral to the stimulus and (b) ipsilateral to the stimulus. The plots correspond to the source/detector pair L-2 and R-2, respectively. The profile of five consecutive trains of stimuli is evident in the contralateral hemisphere, whereas the ipsilateral hemisphere shows no significant changes in CBF.

In each stimulation experiment, the strongest signal was obtained from the second pair (L-2 or R-2) except for one animal, where the signal was better in L-3 and R-3 source–detector pairs. In Fig. 4, simultaneously measured rCBF change from both hemispheres is shown for a representative animal. The cortex contralateral to the stimulus showed changes in rCBF, whereas the ipsilateral cortex results remained unaltered.

**Fig. 5:**
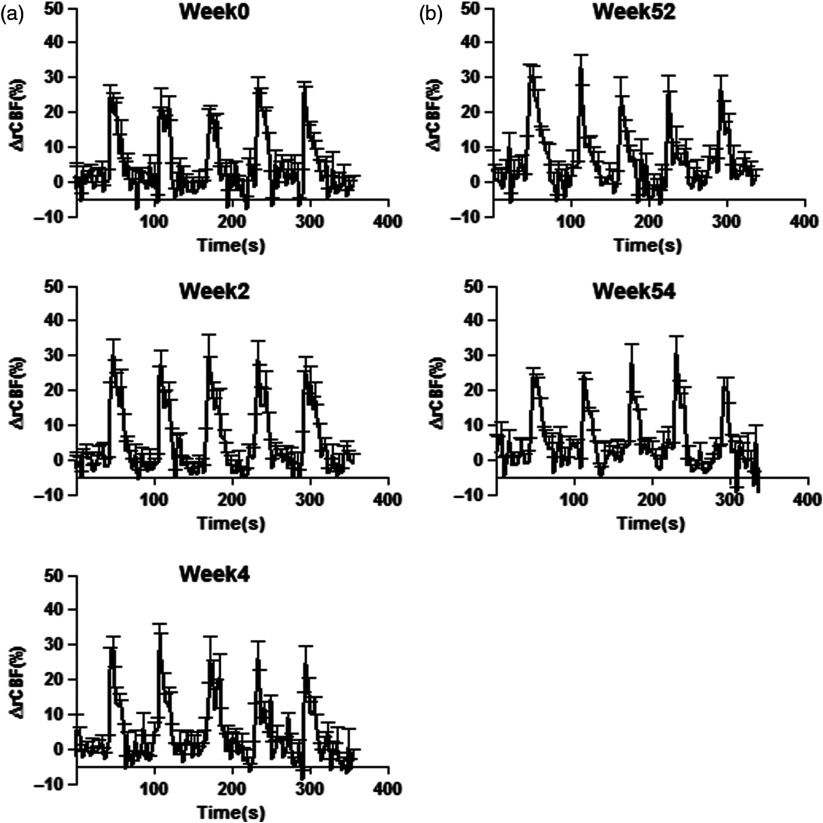
The complete set of experimental sessions from a representative animal. Each plot is composed of six experiments of forepaw stimulation (three right forepaw and three left forepaw). (a) Sessions at the age of 6 month (adulthood, week 0), with a rest period of 2 weeks between sessions (weeks 2 and 4), and (b) sessions at 18 month (elderly, weeks 52 and 54). Changes in CBF measured by DCS did not show statistical significant differences over time (one-way ANOVA; p<0.05).

Figure 5 shows five different sessions from a representative animal, three sessions at the age of 6 months (week 0, week 2, and week 4), and two at the age of 18 months (week 52 and week 54). The study was performed with a resting period of 2 weeks between each experiment cluster. Each graph shows changes in rCBF averaged from six consecutive experiments (three right and three left forepaw stimulation), from L-2 and R-2 source–detector pairs and are represented as mean±SEM. Relative CBF was (22.4±3.8)%; (27.9±5.8)%; and (26.7±6.1)% for week 0, week 2, and week 4, respectively, at the age of 6 months, and (25.8±5.9)%; (24.6±5.4)% for the last sessions at the age of 18 months. Statistical analysis [one-way analysis of variance (ANOVA), p<0.05] showed no significant difference between the sessions from the same animal. In Table 1, we show data for individual weeks and animals, averaged over all six consecutive experiments. The variability in the fMRI signal is 2% to 4%^38^ and in the optics, the variation in the signal is around 10% in accordance with the literature.^26^^,^^39^

**Fig. 6:**
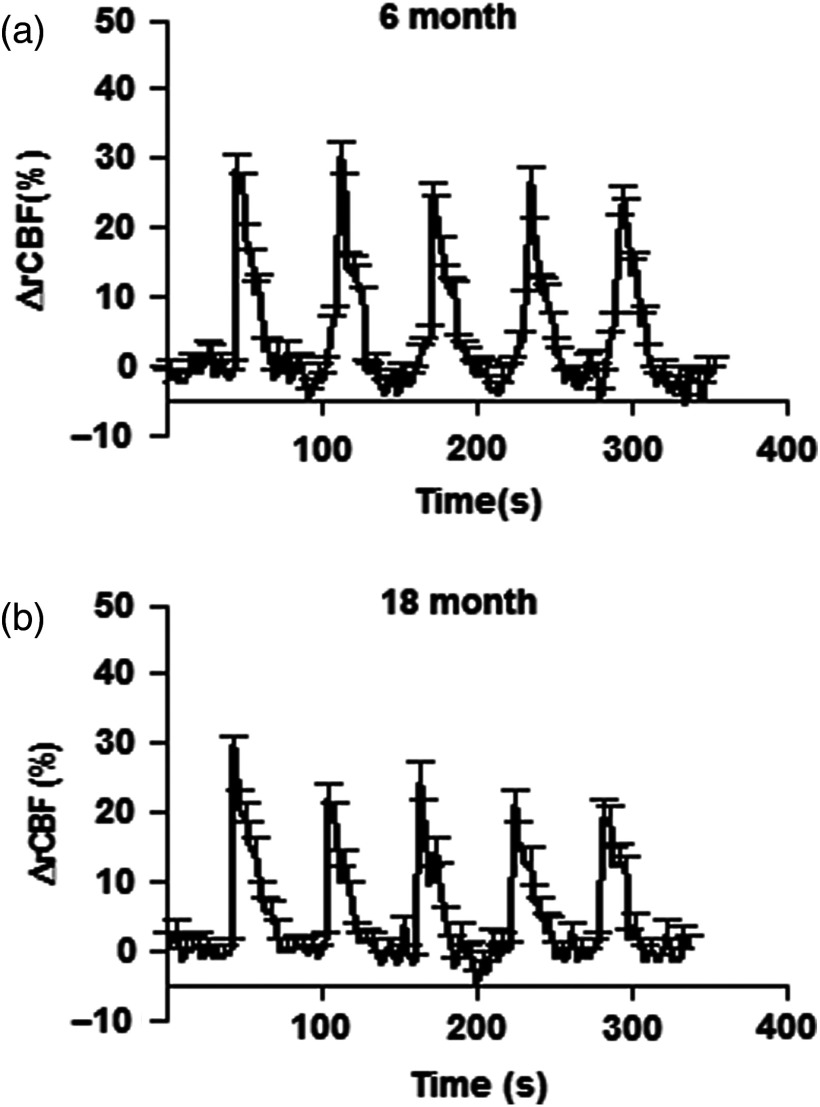
Changes in rCBF (averaged from eight rats), induced by functional brain activity, measured in adulthood (6 months) and in old age (18 months). The percentage change in rCBF did not show significant difference (unpaired t-test; p<0.05).

In order to look at the reproducibility of results in the long-term study, the signal was averaged over L-2 and R-2 source–detector pairs (except for one rat, where the signal was stronger for L-3 and R-3), followed by the intrasubject average for all rats and grouped by age. These results give a similar profile of activation at adulthood and after aging (Fig. 6). The averaged mean of ΔrCBF in the 6 months group was (21.7±3.2)% and 1 year later was (19.5±2.8)% in the same animals. Statistical analysis did not show significant differences between groups (p<0.05).

**Fig. 7:**
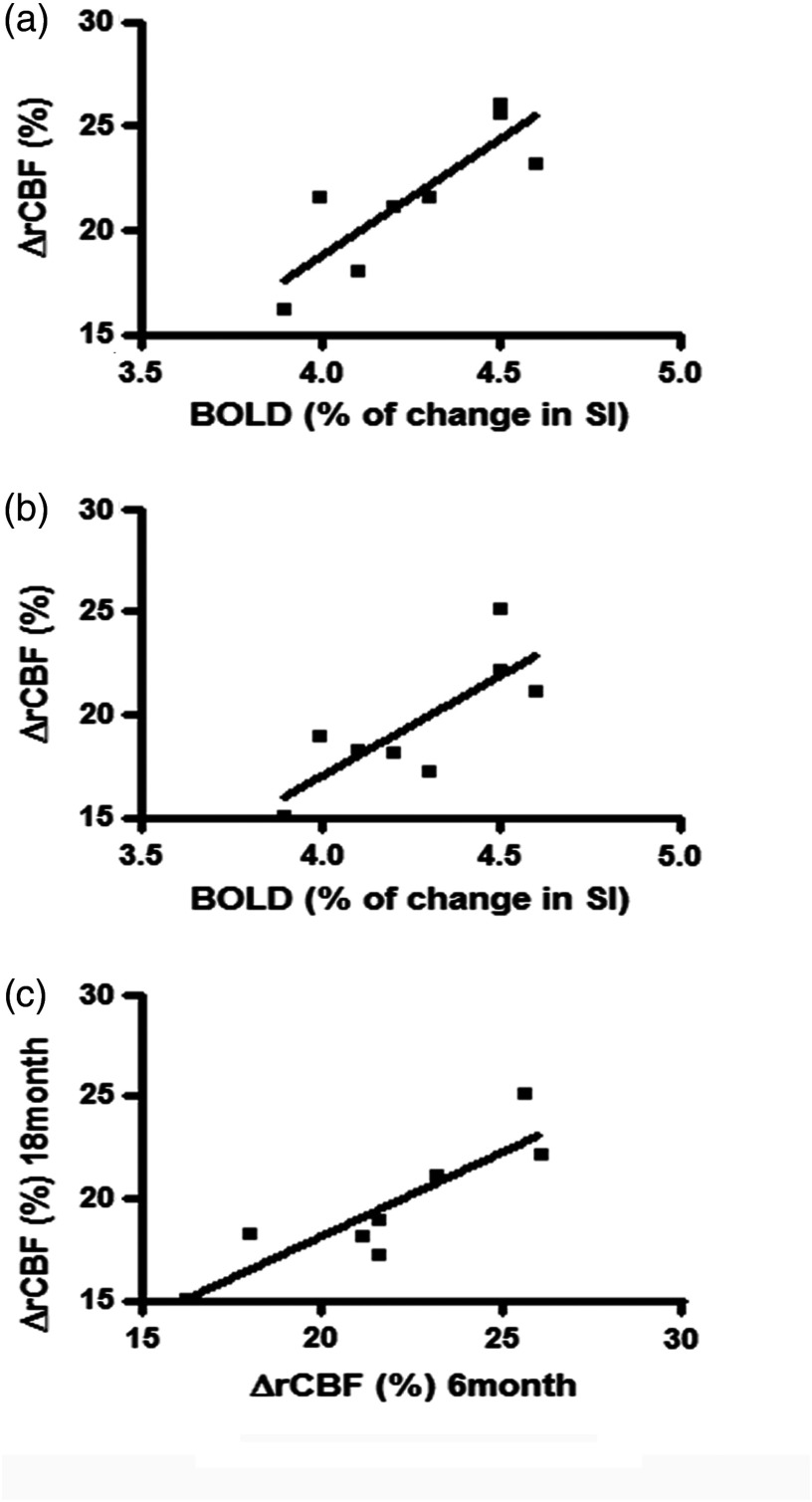
Linear correlation analysis between BOLD and DCS measures as well as between DCS measures at two time-points. (a) ΔrCBF at 6 months is positively correlated to changes in BOLD signal intensity (Pearson’s correlation $r^{2}=0.7$, p<0.05). (b) ΔrCBF at 18 months is positively correlated to BOLD (%SI) with Pearson’s correlation of $r^{2}=0.6$, and p<0.05). (c) Correlation between ΔrCBF (%) at 6 and 18 months ($r^{2}=0.8$, p<0.05).

Linear regression (Fig. 7) shows a positive correlation with the squared Pearson’s correlation coefficient ($r^{2}$) of 0.7 between the BOLD signal and ΔrCBF at the age of 6 months [Fig. 7(a)] and 0.6 at the age of 18 months [Fig. 7(b)] with the p<0.05 for both cases. The correlation between ΔrCBF at 6 and 18 months is shown in Fig. 7(c) with $r^{2}=0.8$ and p<0.05.

## 4 Discussion

The present work demonstrates that DCS is a useful tool to study functional brain activity longitudinally, from a few weeks up to a year, in a noninvasive manner in rats. The data obtained by the transcutaneous DCS probe were reliable and reproducible. The results were comparable to fMRI.

Although DCS has been used in the past to measure tissue blood flow and its alterations in animal models of brain-related pathologies, such as stroke^32^^,^^37^ or postnatal hypoxia,^40^ it has not been used to monitor changes in blood flow longitudinally.

To cross-validate our system, we have compared DCS measurements with fMRI, a well-characterized technique to measure brain activity. DCS measures changes of CBF in the microvasculature,^14^ while fMRI (BOLD) is sensitive to local changes in deoxyhemoglobin concentration produced by the hemodynamic response to a stimulus.^41^^,^^42^ Although DCS and fMRI measure different parameters, both provide similar information about hemodynamic activity reflecting the neurovascular coupling, and are a good indicator of the hemodynamic response promoted by neuronal activity in noninvasive manner.

Most of the methods in optics measuring CBF, such as LDF^21^^,^^22^^,^^43^, LSI,^23^^,^^24^ or OISI,^26^ require the removal of the scalp or/and thinning of the skull to avoid the possibility of masking effects from external tissues on the measurements, or to increase the SNR. All these surgical interventions carry a risk of infection and/or inflammation, which can cause changes in the vasculature of the extracranial tissues as well as the formation of the scar tissue. It can also lead to localized inflammation of the area of the brain closest to the thinned skull because of the heating generated by the drill.^44^ Any of these reactions can cause changes locally in the CBF. In addition, one of the targets of the small animal experiments is to obtain data comparable to those obtained in patients with whom it is generally not possible to use invasive probes.

DCS has been validated in different animal models to measure rCBF^27^^,^^45^ under different anesthesia protocols,^46^ but to our knowledge, no studies have been done in rats with the intact scalp and skull to perform longitudinal studies over months.

Hypoxia, stroke, some neurodegenerative diseases, among other pathologies, cause chronic alterations in brain function. Thus, experimental animal models that mimic such pathologies need to be studied in a longitudinal manner, from days, weeks up to years. In addition, many therapeutic strategies can present their effects in a gradual way, promoting small functional improvements that require a follow-up over an extended time-period. Furthermore, longitudinal studies allow tracking of individual subjects over time, rather than relying on cross-sectional studies with multiple individuals at different time points. In this work, we have shown results on eight rats measured repeatedly at the adult age (6 months old), and the same animals were measured 1 year later (18 months old).

Our experimental paradigm consisted of electrical forepaw stimulation performed under sedation. The optimal neuronal response to nonpainful forepaw electrical stimulation was detected at a stimulating frequency of 6 Hz and is in agreement with previous publications,^47^ thus, DCS experiments were performed at the same frequency, yielding an easily detectable signal. Both BOLD and rCBF results agree with published results in the literature.^44^^,^^48^^,^^49^

In experimental studies with animals, it is essential to use anesthetics or sedatives that immobilize the animal to avoid motion artifacts, reduce stress, and to increase reproducibility.^50^^,^^51^ The most frequently used inhalatory anesthetics agents (halothane, isoflurane) can interfere with normal neural activity, and can affect the neurovascular coupling.^44^^,^^50^^,^^52^^,^^53^ Other anesthetics that preserve such activity often require the use of paralyzing agents, with the need for intubation and mechanical ventilation of the rat, and often with certain degree of toxicity, thus making longitudinal studies impossible. In the last decade, different methods for anesthesia/sedation have been implemented that allow performing functional experiments in a noninvasive and longitudinal manner.^44^^,^^48^^,^^54^

In the present work, a widely validated sedation system for fMRI has been used.^44^^,^^48^^,^^49^ Medetomidine, an α2-adrenoreceptor agonist, produces analgesia, sedation, muscle relaxation, and anxiolysis. However, this sedative has some drawbacks to be considered: it promotes a decrease in the respiratory rate, which may alter the basal levels of CBF and CBV,^44^ and an increase in variability among different individuals due to the depth of sedation. Stress level at the onset of the experiment could make the achievement of an optimal sedation more difficult. Therefore, some animals were excluded from the study. However, all those individuals who achieved satisfactory sedation showed significant changes in CBF evoked by forepaw stimulation. New anesthetics, such as those used recently in fMRI^55^ that minimally alter physiological parameters or neurovascular coupling, could be used in future studies.

The depth penetration in DCS depends on the source–detector separation on the tissue surface. Our probe was designed to detect CBF in the rat cortex at roughly three different depths for both hemispheres [Fig. 2(b)]. The best signal was achieved with the second source–detector pair combination (5.5 mm), which probes a depth of around 2 mm from the surface, except in one session, where the best response was with the third source–detector pair (9.1 mm) for one animal. If we consider the thickness of the scalp and skull, the actual depth of scanning corresponded to the cortex, in agreement with the activation zone in the somatosensory cortex detected by fMRI. The number of source–detector pairs in our probe was limited to improve the temporal resolution. In the future, probes could be implemented by increasing the number of sources and detectors, encompassing a greater area and depth in the cerebral cortex, allowing the composition of accurate activation maps.^14^^,^^28^^,^^31^^,^^56^^,^^57^

We have shown the reproducibility of the results, with little variability when comparing the same individual at different time points, or when we compare between different individuals. Despite this, a certain degree of variability in CBF measurements should be mentioned. The contribution of extracerebral tissues to the measured CBF over time should be considered. Aging increases the skin thickness and the fat content in soft tissues throughout the duration of the experiment (∼1 year). Alterations in the mechanism of neurovascular coupling can take place with aging, due to effects, such as the thickening of the vessel wall and a decrease in elasticity.^58^ Other factors related to age (hypertension, diabetes) have to be taken into consideration when performing longitudinal studies regardless of the pathology of interest. In the future, we believe that with the transcutaneous DCS, it will be possible to study aging and neurovascular coupling in a noninvasive manner in elderly animals.

Furthermore, the case of a slight offset in placement with the transcutaneous probe from the correct place may cause differences in the measured CBF values, since electrical forepaw stimulation elicits a discrete hemodynamic response in a small region of the cerebral cortex. Therefore, the accurate placement of the probe is an issue to consider. External landmarks, such as the position of the eyes, ears, and nose, have been used to localize Bregma as a reference point for the probe placement. However, these landmarks may vary slightly among individuals, as well as with head growing with aging, which may add a component of variability to the measurements.

There is still space for more improvements. We note here that we have followed the literature and used for absorption $0.1\;{\mathrm{cm}}^{-1}$ and for reduced scattering coefficients $15\;{\mathrm{cm}}^{-1}$.^30^ On the other hand, it has been shown previously in the literature^59^ that errors in baseline optical parameters minimally affect high relative changes in CBF especially since the reduced scattering coefficient does not change significantly due to functional stimuli. Furthermore, the current study was a proof-of-principle to show that we can see the changes in the CBF over a long time (1 year). It is possible to use more complex systems (tomography) or models, but those would require different protocols and probes. In the future, measuring changes with a new method SCOT^28^ would give advantages by measuring the CBF through the intact scalp with a large number of detectors.

The used algorithm assumes the homogeneous background of the medium, which may be problematic in the case of high absorptions and for small source–detector separation. Higher order approximations or Monte Carlo models can provide more accurate estimation of the light transport in the tissue,^60^ introducing the heterogeneities for the upper layers, such as the skull.

## 5 Conclusions

The present work demonstrates the ability of DCS to detect neuronal activity through the intact scalp of the rat in a longitudinal manner (over months). This methodology could be useful for the study, in animal models, of the alterations in neuronal activity, which can be compromised in several cerebral pathologies.
